# Students’ Perceptions of Teachers’ Corrective Feedback, Basic Psychological Needs and Subjective Vitality: A Multilevel Approach

**DOI:** 10.3389/fpsyg.2020.558954

**Published:** 2020-10-08

**Authors:** Argenis P. Vergara-Torres, José Tristán, Jeanette M. López-Walle, Alejandra González-Gallegos, Athanasios (Sakis) Pappous, Inés Tomás

**Affiliations:** ^1^School of Sports Organization, Autonomous University of Nuevo León, San Nicolás de los Garza, Mexico; ^2^School of Sport and Exercise Sciences, University of Kent, Gillingham, United Kingdom; ^3^Faculty of Psychology, University of Valencia, Valencia, Spain

**Keywords:** teacher, corrective feedback, physical education, self-determination theory, psychological well-being

## Abstract

The way students perceive corrective feedback has repercussions on what they learn and think. Based on the self-determination theory, the aim of this study is to test a model of multilevel mediation that examines the relationships between the perception of corrective feedback with its degree of acceptance (perceived legitimacy) at the team level and the subjective vitality of students at the individual level, mediated by the satisfaction of the three psychological needs, in the context of physical education. The participants were 742 students aged between 10 and 13 years old (52.6% men, 47.4% women) in 29 physical education groups. The results of the multilevel structural equation modeling analysis found at the group (between) level a positive and significant relationship between corrective feedback and perceived legitimacy (*B*_*between*_ = 0.49, *p* < 0.01), as well as a positive and significant relationship between perceived legitimacy and the needs of competence (*B*_*between*_ = 0.66, *p* < 0.05) and relatedness (*B*_*between*_ = 0.95, *p* < 0.01). In addition, there was a positive and significant association between competence and subjective vitality (*B*_*between*_ = 2.06, *p* < 0.01), and a negative and significant association between relatedness and subjective vitality (*B*_*between*_ = −0.85, *p* < 0.01). Also, on an individual (within) level, the needs of autonomy (*B*_*within*_ = 0.09, *p* < 0.05), competence (*B*_*within*_ = 0.27, *p* < 0.01), and relatedness (*B*_*within*_ = 0.17, *p* < 0.01) were positively and significantly associated with subjective vitality. Finally, corrective feedback showed a positive indirect effect on subjective vitality through perceived legitimacy and competence, while the indirect effect was negative through perceived legitimacy and relatedness. In conclusion, on an individual level, students who perceive their basic psychological needs to be met in turn, increase their subjective vitality. At the group level, the results are discussed. These findings suggest that teachers might be best advised to ensure that their students accept corrective feedback, by having it couched in a manner that suggests that learning and improvement can follow, and communicated in an autonomy-supporting way.

## Introduction

Providing corrective feedback within the physical education class or training is inevitable, as it is inherent to the teaching process (Mouratidis et al., 2010). However, because of its negative nature (for it does not focus on the approval of a performance), it could have undesired repercussions on students (Mouratidis et al., 2010; Tristán et al., 2017, 2018). Therefore, it is necessary for the teacher to create a learning environment that leads students to not perceive it as threatening, challenging, unfair, and/or unjustified. Corrective feedback (Mouratidis et al., 2010; Carpentier and Mageau, 2013, 2016) or structure during the activity (Haerens et al., 2013), is defined as statements that convey information about aspects to be improved after poor performance (Mouratidis et al., 2010; Carpentier and Mageau, 2013, 2016).

The great diversity of students within the physical education class may lead to different outcomes in the way corrective feedback is perceived (Henderlong and Lepper, 2002; Mouratidis et al., 2010; Asún et al., 2020), that is why it is considered that perceived legitimacy, or the degree of acceptance of corrective feedback, is critical to its benefit within a learning environment (Mouratidis et al., 2010; Ríos, 2015; Tristán et al., 2017, 2018). In general, studies of negative feedback (and that have not considered the legitimate perception of the students about it self) have related it negatively to intrinsic motivation (Koka and Hein, 2003; Hollembeak and Amorose, 2005; Koka and Hein, 2006) and perceived competence (Koka and Hein, 2003). Furthermore, it has been observed that negative feedback can have a detrimental effect on students’ self-esteem, self-efficacy and motivation to learn (Kim, 2004; Hattie and Timperley, 2007; Shute, 2008; Voerman et al., 2012).

Recently, one of the theories that has studied the phenomena resulting from teacher-student interaction is the Self-Determination Theory (SDT; Deci and Ryan, 1985; Ryan and Deci, 2017). More specifically, one of its mini-theories, the Basic Psychological Needs Theory (BPNT), which assumes the existence of three basic psychological needs: autonomy (experience of choice and psychological freedom with respect to one’s action), competence (experience of effectiveness in social interactions and learning tasks) and relatedness (feeling of connection with other people and belonging to a group). Those three needs are considered to be essential nutrients for growth, integrity and physical, and psychological health (Deci and Ryan, 2000; Ryan and Deci, 2002, 2017). The BPNT also states that the satisfaction of basic psychological needs generates psychological well-being, whose indicator for “excellence” is subjective vitality, defined as the feeling of possessing energy, dynamism and vigor (Ryan and Frederick, 1997). Furthermore, this theory considers that the development of well-being is a function of the social context and its potential to satisfy psychological needs (Deci and Ryan, 2000; Ryan and Deci, 2000, 2002, 2017). From this perspective, in the field of physical education, the teacher can be seen as a social agent, representing a figure of authority and leadership for the students, given that the intervention of a teacher in the classroom plays a determining role in the satisfaction of basic psychological needs (Ntoumanis and Standage, 2009; Wilson et al., 2012; Curran and Standage, 2017; Tristán et al., 2019) and these, in turn, in the students’ perceptions of vitality (Tristán et al., 2019).

Despite this, the literature has not sufficiently delved into corrective feedback, students’ legitimate perception (perceived legitimacy) and their relation to the satisfaction of basic psychological needs in the context of physical education, even though according to SDT, basic psychological needs mediate students’ motivation and psychological well-being. It is therefore essential to understand how the teacher should communicate corrective feedback so that it is perceived by students in a legitimate way and can meet their needs of autonomy, competence and relatedness, for it is possible that their perception of corrective feedback in the physical education session, regardless of whether it conveys a message of low competence, does not generate feelings of incompetence, low autonomy and poor relatedness, and does not undermine the intrinsic motivation of students to continue with task practice.

There is some indirect support for this position in the literature, although studies have been conducted in sports settings (Mouratidis et al., 2010; Carpentier and Mageau, 2013, 2016; Ríos, 2015; Tristán et al., 2017, 2018). The results in these investigations have found that corrective feedback can be positively related to the satisfaction of each of the basic psychological needs (Carpentier and Mageau, 2013, 2016; Ríos, 2015; Tristán et al., 2017), this is to the extent that corrective feedback is given in a style that supports autonomy and is therefore perceived as legitimate by the athletes (Mouratidis et al., 2010; Ríos, 2015; Tristán et al., 2017, 2018). It has also been found that when corrective feedback is conveyed in a friendly, tolerant, respectful and understanding manner, athletes experience higher levels of intrinsic motivation and autonomy (Mouratidis et al., 2010; Carpentier and Mageau, 2016), self-esteem (Carpentier and Mageau, 2013), self-confidence (Carpentier and Mageau, 2016), and greater subjective vitality (Ríos, 2015; Carpentier and Mageau, 2016).

Although sport and physical education classes share many elements in common, they also have different purposes and means to achieve them (Wallhead and O’sullivan, 2005), which is why testing the phenomena that occur in sport in physical education also becomes relevant. Furthermore, it is necessary to take into account that the benefits that could be derived from participation in physical education classes do not occur automatically, but depend on the quality of teacher-student interactions (Bailey, 2006). In this sense, the provision of corrective feedback within the physical education class becomes a way to improve and self-regulate student learning (Contreras-Pérez and Zuñiga-González, 2017), thereby becoming a very important aspect of teacher intervention.

Together with this, the physical education class represents the only space in which many children and teenagers can carry out physical activity (Villagrán et al., 2010; Abarca-Sos et al., 2015). In consequence, studying the aspects that intervene in the physical and psychological health of the students takes importance. Despite the fact that the model hypothesized in this work has been tested in the context of sports (Ríos, 2015), so far, there is a gap in the literature about the consequences that corrective feedback might have on the satisfaction of psychological needs and on the perceptions of vitality within the physical education class.

On the other hand, although the variables involved in this study (corrective feedback, legitimate perception, basic psychological needs, and subjective vitality) have been theorized as individual constructs, the coexistence, collaboration, participation, and playing that takes place among the students within the classes could generate different perceptions from a group perspective, in other words, the perception of a group/team, may vary from the individual perspective of each of the students. Which is why it is also necessary to analyze the multilevel perspective, as previous studies have done (e.g., Papaioannou et al., 2004; Wilson et al., 2012; Beauchamp et al., 2014).

**FIGURE 1:**
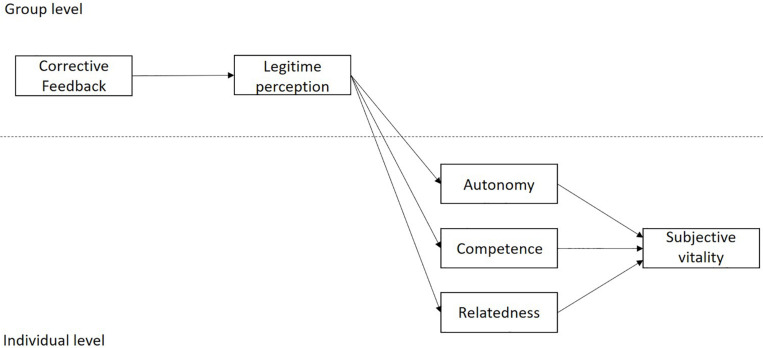
Graphical representation of the hypothesized conceptual model.

For all these reasons, the main contributions of this study to the previous literature are: (1) to test the hypothesized model in the context of the physical education class, (2) to bring the multilevel approach into the study of these relationships. Therefore, the general objective of the study is to test, in the context of physical education, a model of multilevel mediation (see Figure 1) that examines the relations between the variables of corrective feedback, the degree of acceptance of corrective feedback (legitimate perception) at the group level and the subjective vitality of students at the individual level, mediated by the satisfaction of the three psychological needs. In addition, the following hypotheses have been put forward: (1) corrective feedback will have an indirect effect on subjective vitality through the legitimate perception and satisfaction of the need for autonomy; (2) corrective feedback will have an indirect effect on subjective vitality through the legitimate perception and satisfaction of the need for competence and, (3) corrective feedback will have an indirect effect on subjective vitality through the legitimate perception and satisfaction of the need for relatedness.

## Materials and Methods

### Procedure and Participants

The principals of the selected schools were contacted through a letter explaining the purpose of the study and requesting authorization for the administration of the instruments by providing them with a copy of the instruments. Due to the multilevel approach of this study, all students in the sixth-grade groups of the participating schools were considered. The selection criteria were that the groups should have at least one physical education class per week taught by a physical education professional, while at the individual level it was established that the participant was a regular student at the school. The exclusion criteria were: that the student had a cognitive disability that prevented him/her from answering the questionnaire consciously/autonomously. The signature of the informed consent letter was requested from the student’s parent or guardian. Only those students who submitted informed consent participated in the study. The questionnaires were answered anonymously between May and June 2019, under the supervision of a researcher in a classroom during a school day, and without the presence of the physical education teacher. The purpose of the research was explained to the students as well as the voluntariness and confidentiality of the answers and use of the data. They were also informed that there were no correct or incorrect answers and they were asked to answer honestly. In order to guarantee homogeneity in the conditions of data collection, the interviewers were previously trained. Data collection procedure followed the ethical standards recommended by the American Psychological Association (APA).

Due to the complexity to cover the large number of schools in the metropolitan area of Monterrey, Mexico, that could be selected in a representative sample, a convenience sample was chosen, having as a common variable in all the selected schools being from areas with medium and low-medium socioeconomic status.

The participants were 742 students (52.6% men, 47.4% women) and 29 physical education groups/class from public primary schools, located in the metropolitan area of Monterrey, México. The organization of the groups/classes carried out by the schools before the beginning of the school year was taken, so the authors did not intervene in the organization of these groups. The age range of the students was between 10 and 13 years old (*M* = 11.35, *SD* = 0.49) and had one or two physical education classes per week (*M* = 1.57 and *SD* = 0.40). Classes duration were between 40 and 60 min each class (*M* = 45.43, *SD* = 5.47).

Regarding the power analysis, this sample should have sufficient statistical power to detect relevant relationships at the individual level. According to sample size and statistical power calculations in multiple regressions, assuming a low effect size (f^2^ = 0.05) for a maximum number of predictors (5) and an alpha level of 0.05, in order to attain a statistical power level of 0.80, the required sample size would be 263 (Faul et al., 2009). The study sample at the individual level was composed of 742 students, thus, larger than the required to attain an adequate power level.

Considering the group level of analysis, previous literature indicate that a statistical power of at least 0.80 can be obtained with samples of about 30 groups with around 20 members (Bell et al., 2014). In this study, the multilevel analysis were run with 742 students belonging to 29 physical education groups, with a group average size of 25.6 students per group.

### Measures

To measure perceptions of corrective feedback received by the students and their perceived legitimacy, an adapted version to the physical education context of the Amount of Corrective Feedback Scale in sport was used (Tristán et al., 2013). This scale is made up of four subscales with four items each (16 items in total): corrective feedback scale, legitimate perception, opportunity to learn, and illegitimate perception. For the purpose of this study, only the corrective feedback and perceived legitimacy subscales were used. All items were answered using a Likert response scale in a range of 1 (*completely disagree*) to 5 (*completely agree*). An example of an item in the corrective feedback subscale is: “*Is it true that your physical education teacher points out mistakes?*” In the perceived legitimacy subscale, an example of an item is: *“If my teacher points out my mistakes, I find that he (she) has a fair reason to do so.”*

To assess the satisfaction of basic psychological needs, the Mexican Scale of Satisfaction of Basic Psychological Needs in Physical Education was used, adapted and validated by Zamarripa et al. (2017). This instrument presents three subscales that measure the needs of autonomy, competence and relatedness through a total of 16 items with a Likert-type response scale of seven points, in a range of 1 (*strongly disagree*) to 7 (*strongly agreement*). To measure the need for autonomy, the instrument uses six items headed by the statement: “In this physical education class ….” An example of an item in this subscale is *“my opinion counts as to what activities I want to practice.”* For the need for competence, the corresponding subscale is made up of five items, preceded by the heading: *“In this physical education class…,”* and an example of an item is: *“I think I’m pretty good.”* Finally, to assess the need for relatedness the subscale is made up of five items preceded by the statement: *”With the other students in my physical education class I feel…”* and where an example of a response is: *“understood.”*

Subjective vitality was measured through an adaptation to the context of physical education of the Spanish Subjective Vitality Scale in its Mexican version (López-Walle et al., 2012; Castillo et al., 2017). This scale is composed of six items with a Likert response scale, whose range is from 1 (*not true*) to 7 (*true*). An example of an item is *“I feel encouragement and enthusiasm (alive) and full of life (vital).”*

The adaptations of the instruments were carried out by a panel of experts in physical education as well as active physical education teachers. The instruments were also piloted with reduced samples of primary level physical education students to verify the adequate compression of the items.

### Data Analysis

Descriptive analyses were performed for each of the study variables (mean, standard deviation, skewness and kurtosis). The normality of the data was determined following Muthén and Kaplan (1985, 1992) and Ferrando and Anguiano-Carrasco (2010), who recommend coefficients of skewness and kurtosis in a range of -1, 1. The Pearson correlation test was carried out to analyze the interrelations between the variables and establish the level of association between them. To evaluate the reliability of the scales used, internal consistency analyses were performed using Cronbach’s alpha as an indicator. Descriptive and correlation analyses were performed using SPSS 25.0 software.

To assess the factorial structure of the hypothesized model, confirmatory factor analysis (CFA) was carried out using the Maximum Likelihood (ML) as the estimation method. The model fit indices used were chi-square (χ^2^), the Root Mean Square Error of Approximation (RMSEA), the Comparative Fit Index (CFI), the Tucker Lewis Index (TLI), and the standardized root mean square residual (SRMR) (Hu and Bentler, 1999; Kline, 2005).

In order to justify data aggregation for the two variables considered at the team level (corrective feedback and legitimate perception), within-group agreement, interrater reliability and between group discrimination were tested. To evaluate within-group agreement, several indices were estimated: the Average Deviation Index (ADI, Burke et al., 1999), the *r*_*WG(J)*_ (James et al., 1984), and the intraclass correlation coefficients ICC1 (Bliese, 2000). The following cut-off values were used as indicators of within-group agreement: ADI values below 0.83 for a 5-point Likert response scale (Burke and Dunlap, 2002), ICC1 values higher than 0.05 (LeBreton and Senter, 2008), and *r*_*WG(J)*_ values above 0.70 (Bliese, 2016). ICC2 was used to estimate the reliability of the group mean (interrater realibility), with values above 0.70 considered as satisfactory (Bliese, 2000). To determine the existence of statistically significant discrimination between groups, one-way analysis of variance (ANOVA) was used.

Finally, to assess the hypothesized structural model, multilevel structural equation modeling (MSEM) was used with Mplus software (Muthén and Muthén, 1998–2010), using maximum likelihood estimation. According to Zhang et al. (2009), in this study the proposed model was a 2-2-1-1 model, where corrective feedback and perceived legitimacy were level two variables, and basic psychological needs satisfaction (autonomy, competence, and relatedness), and subjective vitality were level one variables (see Figure 1). To test the significance of the indirect effects, the Monte Carlo (MC) confidence interval (CI) method was used, using the web estimator provided by Selig and Preacher (2008). This method has been suggested to determine indirect effects in multilevel models (Preacher and Selig, 2012).

## Results

### Descriptive Statistics

Table 1 presents the descriptive statistics of the variables of the study. Skewness and kurtosis values, followed a normal distribution. Cronbach alpha values were above 0.70, except for autonomy which showed a value of 0.68. Thus, according to Peterson (1994), the reliability level of the whole scale is to be considered satisfactory, with the exception of the autonomy subscale which presented an adequate degree of reliability. Additional reliability analyses were run for the autonomy subscale. Concretely, the Omega coefficient (McDonald, 1999) and the composite reliability value (Rho) (Raykov, 2001) were estimated, which turned out to show adequate values (Omega = 0.75, rho = 0.75). Considering these results, and taking into account the theoretical relevance of the variable, it was decided to maintain the autonomy subscale in the model. Finally, all the correlations between the analyzed variables were positive and statistically significant (*p* < 0.01) with exception of the relationship between group legitime perception and autonomy.

**TABLE 1:** Descriptive statistics, reliability, and correlation between study variables.

	***M***	***SD***	**α**	**Skewness**	**Kurtosis**	**1**	**2**	**3**	**4**	**5**
1 Group corrective feedback	3.90	0.25	0.76	−0.25	–0.25	–				
2 Group legitime perception	4.27	0.20	0.73	−0.47	0.02	0.63**	–			
3 Autonomy	4.47	1.17	0.68	−0.16	–0.45	0.19**	0.03	–		
4 Competence	5.16	1.19	0.81	−0.58	0.36	0.15**	0.12**	0.36**	–	
5 Relatedness	5.44	1.33	0.92	−0.73	0.18	0.14**	0.15**	0.39**	0.55**	–
6 Subjective Vitality	5.14	1.11	0.79	−0.61	–0.08	0.20**	0.10**	0.27**	0.46**	0.43**

M, Mean; SD, Standard deviation; α, Cronbach’s alpha; **p < 0.01.

### Confirmatory Factor Analysis

Two alternative CFA models were tested: a four factors model (corrective feedback, legitimate perception, basic psychological needs satisfaction and subjective vitality), and the hypothesized six factors model (corrective feedback, perceived legitimacy, need for autonomy, need for competence, need for relatedness, and subjective vitality).

The four factors model showed non-satisfactory fit to data [χ^2^(397) = 1644.56, χ^2^*/gl* = 4.142 *p* < 0.001; RMSEA = 0.06; CFI = 0.82; TLI = 0.80; SRMR = 0.06], while the hypothesized six factors model showed a satisfactory fit to data [χ^2^(390) = 875.46, χ^2^*/gl* = 2.224 *p* < 0.001; RMSEA = 0.04; CFI = 0.93; TLI = 0.92; SRMR = 0.04]. Additionally, all the items in the six factors model showed statistically significant factor loadings in their corresponding factors (*p* < 0.01).

### Justification of Data Aggregation

The ADI value for corrective feedback was 0.78 (*SD* = 0.19) and for perceived legitimacy was 0.64 (*SD* = 0.11). The *r*_WG(J)_ value for corrective feedback was 0.74 and for perceived legitimacy was 0.87. The ICC1 value for corrective feedback was 0.063 and for perceived legitimacy was 0.069. All these values together indicated that there was within-group agreement on perceived corrective feedback and perceived legitimacy. The ICC2 value for corrective feedback was 0.63 and for perceived legitimacy was 0.65, indicating acceptable interrater reliability (Bliese, 2000). Finally, the ANOVA results for corrective feedback [*F*_(28, 713)_ = 2.747, *p* < 0.001] and perceived legitimacy [*F*_(28, 713)_ = 2.914, *p* < 0.001] indicated statistically significant differences between the groups in perceived corrective feedback and perceived legitimacy. According to these values, justification for data aggregation at the group level for corrective feedback and perceived legitimacy was supported, and thus, to test the proposed model using multilevel structural equation modeling.

### Multilevel Structural Equation Modeling

The hypothesized multilevel model presented adequate goodness of fit indices [χ^2^(5) = 16.898, χ^2^*/gl* = 3.38 *p* < 0.01; RMSEA = 0.057; CFI = 0.98; TLI = 0.92; SRMR within = 0.001, SRMR between = 0.16].

**FIGURE 2:**
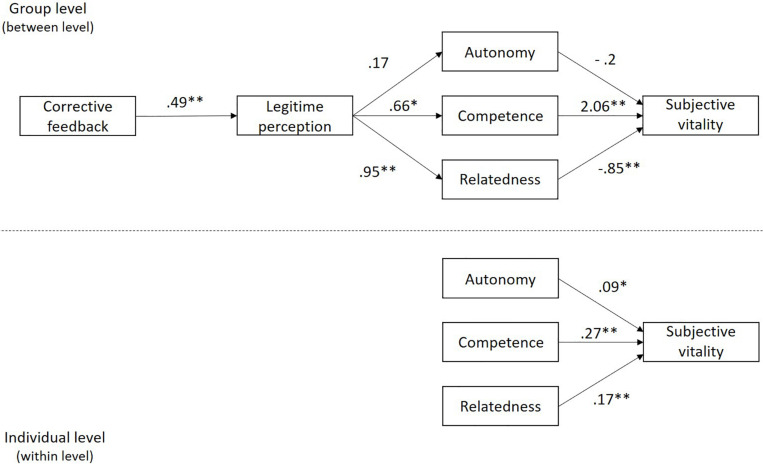
Non-standardized results of the hypothesized model. *p < 0.05; **p < 0.01.

Figure 2 shows the results of the multi-level mediation model. First, at the group level (between level), a positive and statistically significant relation (*B* = 0.49, *p* < 0.01) can be observed between corrective feedback provided by the teacher and perceived legitimacy. A positive but not significant relation (*B* = 0.17, *p* > 0.05) between perceived legitimacy and psychological need satisfaction of autonomy. A positive and significant relation between perceived legitimacy and psychological needs satisfaction of competence and relatedness (*B* = 0.66, *p* < 0.05; *B* = 0.95, *p* < 0.01, respectively), as well as a negative and not significant association between psychological need of autonomy and subjective vitality (*B* = −0.20, *p* > 0.05). A positive and statistically significant relation between psychological need satisfaction of competence and subjective vitality (*B* = 2.06, *p* < 0.01), and finally, a negative and significant association between psychological need satisfaction of relatedness and subjective vitality (*B* = −0.85, *p* < 0.01).

Additionally, at the individual level (within level), results showed a positive and statistically significant relation between basic psychological needs satisfaction (autonomy, competence, and relatedness) and subjective vitality (*B* = 0.09, *p* < 0.05; *B* = 0.27, *p* < 0.01; *B* = 0.17, *p* < 0.01, respectively).

Regarding the between level indirect effects, corrective feedback showed a positive and significant relationship with subjective vitality trough perceived legitimacy and psychological need satisfaction of competence (unstandardized estimate of the indirect effect = 0.67, *p <* 0.05, 95% MC CI = 0.23, 0.36); moreover, corrective feedback showed a negative and significant relationship with subjective vitality trough perceived legitimacy and psychological need satisfaction of relatedness (unstandardized estimate of the indirect effect = −0.4, *p <* 0.05, 95% MC CI = −0.86, -0.1). Those results allowed concluding that perceived legitimacy and the satisfaction of the basic psychological needs of competence and relatedness, mediated the relationship between corrective feedback and subjective vitality.

Finally, in order to know the type of mediation of perceived legitimacy and the satisfaction of basic psychological needs (competence and relatedness) in the relationship between corrective feedback and subjective vitality, an alternative model with a direct relationship between corrective feedback and subjective vitality was tested. The fit of the partial mediation model was adequate [χ^2^(4) = 10.029, χ^2^*/gl* = 2.507 *p* < 0.05; RMSEA = 0.045; CFI = 0.99; TLI = 0.95; SRMR within = 0.001, SRMR between = 0.15]. When comparing the partial mediation model with the hypothesized full mediation model, the differences between the two nested models on RMSEA were non-relevant (ΔRMSEA = 0.012), but ΔTLI and ΔCFI values (ΔTLI = 0.029; ΔCFI = 0.010) showed relevant differences, indicating that the alternative partial mediation model was the best fitting model. Moreover, the relationship between corrective feedback and subjective vitality was significant (*B* = 0.67, *p* < 0.05). Thus, partial mediation was supported.

## Discussion and Conclusion

The general aim of this study was to test, under the postulates of the Self-Determination Theory (Ryan and Deci, 2017), a model that examined the relations between corrective feedback, legitimate perception at the group level and subjective vitality at the individual level, mediated by the satisfaction of basic psychological needs. Although a similar model has already been tested in the field of sports (Ríos, 2015; Tristán et al., 2017), it had not been applied in physical education and had not been approached from a multilevel perspective.

Starting from the group level, a positive and significant association was observed between corrective feedback and the perceived legitimacy of physical education groups. Up to the time of the writing of this manuscript, there are no known studies at the group level that analyze these two variables. Nevertheless, this finding is similar to that reported in previous studies at the individual level in the sports context (Mouratidis et al., 2010; Ríos, 2015; Tristán et al., 2017), and therefore, just as in the trainer-athlete relationship (Mouratidis et al., 2010; Carpentier and Mageau, 2013, 2016; Ríos, 2015; Tristán et al., 2017, 2018), physical education students also perceive their teacher’s corrections as fair and reasonable (legitimate) when they are given in an autonomy-supportive style.

Similarly, it was found at group level that legitimate perception was positively and significantly associated with competence and relatedness needs, an association that also occurs at individual level in the field of sports (Ríos, 2015; Tristán et al., 2017). Therefore, in the physical education class, it is also important for teachers to ensure that their students have a high degree of acceptance of the corrective feedback they have provided, and that their competence and relatedness needs are met. However, the association between legitimate perception and the need for autonomy, contrary to the sports context (Ríos, 2015; Tristán et al., 2017), was not significant. This could be explained by the fact that students do not perceive a sense of choice and freedom about how to act to correct their mistakes (Taylor et al., 2010), and those perceptions of autonomy are more likely to fluctuate depending on the context (Cox et al., 2008).

In addition to the above, also at a group level, and contrary to what has been hypothesized in this work, and to what has been reported at an individual level in the field of sports (Adie et al., 2008; López-Walle et al., 2012; Ríos, 2015; Garza-Adame et al., 2017), as well as in physical education (Taylor and Lonsdale, 2010; Tristán et al., 2019), the need for autonomy was not positively or significantly related to subjective vitality, which was the case for the need for competence and was also the strongest relation found in the proposed model. On the other hand, the least expected result was the association between the need for relatedness and subjective vitality, which was negative and significant. These results seem to show that the functional meaning of the need for competence may be greater than the needs of autonomy and relatedness in the field of physical education (Standage et al., 2006). In other words, the physical education groups with a higher satisfaction of their need for competence showed greater subjective vitality (Taylor et al., 2010). In addition, in a study (Wilson et al., 2012) that analyzed at a group level and as a single factor basic psychological needs, these were not significantly associated with ill-being variables (motivation and engagement), which is partly consistent with some results of this study.

Finally, at the individual level, a positive and statistically significant relation was observed between the autonomy, competence and relatedness needs, and subjective vitality, which is similar to that found in sport-related research (Adie et al., 2008; López-Walle et al., 2012; Garza-Adame et al., 2017) and supports the idea that basic psychological needs are more individual constructs (Ryan and Deci, 2017). However, more studies need to be done at the group level to be clearer about the satisfaction of psychological needs and their role on subjective vitality.

Complementarily, in the first hypothesis raised for this work, it was expected to find a positive indirect effect between corrective feedback and subjective vitality through legitimate perception and the satisfaction of the need for autonomy. The results obtained showed a non-significant indirect effect. This result could be related to the negative association between autonomy and subjective vitality presented in this study, because autonomy is more likely to fluctuate according to context and is more strongly derived from situational factors such as social relations with the physical education teacher and classmates (Cox et al., 2008). This result indicates that teachers, when providing corrective feedback, need to do so in an empathetic, accurate and option-oriented manner, as well as providing the opportunity for active participation of students in decision making, in order for them to experience greater autonomy (Carpentier and Mageau, 2013, 2016).

Hypothesis two established that corrective feedback would have a positive indirect effect on subjective vitality through legitimate perception and the need for competence. The results obtained confirmed what was established in the hypothesis, since a positive and significant indirect effect was found among these variables. In that sense, this finding is added to the apparent prominence of the need for competence found at group level in the model analyzed. As mentioned above, in the context of physical education, physical competences are very visible (Taylor et al., 2010), so high perceptions of competence by students, together with legitimate perceptions of corrective feedback provided by the teacher, are associated with higher subjective vitality.

Hypothesis three stated that corrective feedback would have a positive indirect effect on subjective vitality through the need for relatedness, however, the results obtained showed a significant but negative indirect effect, which was contrary to what was expected. This could be explained by the fact that the need for relatedness is more likely to fluctuate according to the context and may derive more strongly from situational factors such as social relations with the physical education teacher and classmates (Cox et al., 2008). This result allows us to point out, after having analyzed each psychological need, that the need for competence may be less sensitive to situational influence (Cox et al., 2008) and publicly more visible and prominent in physical education than the perceptions of autonomy and relatedness (Standage et al., 2006; Cox et al., 2008).

The results obtained in this study represent an important contribution to the literature due to the scarcity of research related to corrective feedback in physical education classes. It is to be notice that corrective feedback in this study has been approached from both a group and individual perspective, and it corroborates what has been suggested in previous studies (Koka and Hein, 2005), where despite of focusing on error, when communicated in an assertive manner, corrective feedback can favor perceived competence.

In addition to the contributions of this study, limitations must also be recognized. Firstly, in comparison to other studies using multilevel analysis (Wilson et al., 2012; Beauchamp et al., 2014; Estreder et al., 2019), this study had participants distributed in few physical education groups, which may have had an impact on representativeness as well as on the power of the test and therefore on not detecting potentially significant relations. Secondly, this study measured whether the student perceived that their teacher provided corrective feedback, but not how often it was provided. Future studies could consider measuring the frequency of perceived corrective feedback, as well as including the level of perception and acceptance among boys and girls. Finally, future studies could further study the corrective feedback received, as well as its legitimate and illegitimate perception and symptoms of well-being/ill-being with a multilevel approach, as well as conducting research with longitudinal designs.

In conclusion, at group level, when physical education students perceive the corrective feedback provided by their teachers as legitimate, they perceive their psychological needs for competence and relatedness to be more satisfied, with only the satisfaction of the need for competence being associated with greater perceptions of vitality. At the individual level, the satisfaction of the need for autonomy, competence and relatedness is associated with increased perceptions of vitality. Therefore, it is important that teachers ensure the acceptance of corrective feedback given to students by adopting an autonomy-supportive style.

## Practical Applications

The results of the present study suggest that for corrective feedback to have a positive impact on physical education students, it must be provided under characteristics that guarantee its acceptance by the students and thus be perceived as more vital, so that there is adequate communication and interaction between the teacher and his/her students. These results can contribute to the development of training programs for in-service and pre-service physical education teachers to make their classroom intervention more effective. Similarly, the suggestions and findings of the present study could be used for the development of teacher performance assessment tools from the perspective of an external evaluator.

## Data Availability Statement

The raw data supporting the conclusions of this article will be made available by the authors, without undue reservation, to any qualified researcher.

## Ethics Statement

The studies involving human participants were reviewed and approved by the Comité de Investigación de la Facultad de Organización Deportiva under registration number: REPRIN-FOD-67. Written informed consent to participate in this study was provided by the participants’ legal guardian/next of kin.

## Author Contributions

JT, APV-T, JML-W, and IT contributed to the conception and design of the study. APV-T, AG-G, and IT organized the database and performed the statistical analysis. APV-T and JT wrote the first draft of the manuscript. APV-T, JT, IT, JML-W, and AP wrote the sections of the manuscript. AG-G and AP global review of the article and relevance of the translation. All authors contributed to manuscript revision, read and approved the submitted version.

## Conflict of Interest

The authors declare that the research was conducted in the absence of any commercial or financial relationships that could be construed as a potential conflict of interest.
